# Newcastle Disease Virus: Potential Therapeutic Application for Human and Canine Lymphoma

**DOI:** 10.3390/v8010003

**Published:** 2015-12-23

**Authors:** Diana Sánchez, Rosana Pelayo, Luis Alberto Medina, Eduardo Vadillo, Rogelio Sánchez, Luis Núñez, Gabriela Cesarman-Maus, Rosa Elena Sarmiento-Silva

**Affiliations:** 1 Faculty of Veterinary Medicine and Animal Husbandry, National Autonomous University of Mexico, Mexico City 04510, Mexico; luisno@unam.mx; 2 Medical Research Unit, Oncology Hospital, Mexican Institute for Social Security, Mexico City 06720, Mexico.; rosanapelayo@gmail.com (R.P.); evadillo@hotmail.com (E.V.); 3 Biomedical Cancer Research Unit, National Cancer Institute and Physics Institute, National Autonomous University of Mexico, Mexico City 14080, Mexico; medina@fisica.unam.mx; 4 Pathology Department, Spanish Hospital, Mexico City 11520, Mexico; genryus_blade@hotmail.com; 5 Hematology Department, National Cancer Institute, Mexico City 14080, Mexico

**Keywords:** Newcastle disease virus, lymphoma, oncolytic virus

## Abstract

Research on oncolytic viruses has mostly been directed towards the treatment of solid tumors, which has yielded limited information regarding their activity in hematological cancer. It has also been directed towards the treatment of humans, yet veterinary medicine may also benefit. Several strains of the Newcastle disease virus (NDV) have been used as oncolytics *in vitro* and in a number of *in vivo* experiments. We studied the cytolytic effect of NDV-MLS, a low virulence attenuated lentogenic strain, on a human large B-cell lymphoma cell line (SU-DHL-4), as well as on primary canine-derived B-cell lymphoma cells, and compared them to healthy peripheral blood mononuclear cells (PBMC) from both humans and dogs. NDV-MLS reduced cell survival in both human (42% ± 5%) and dog (34% ± 12%) lymphoma cells as compared to untreated controls. No significant effect on PBMC was seen. Cell death involved apoptosis as documented by flow-cytometry. NDV-MLS infections of malignant lymphoma tumors *in vivo* in dogs were confirmed by electron microscopy. Early (24 h) biodistribution of intravenous injection of 1 × 10^12^ TCID_50_ (tissue culture infective dose) in a dog with T-cell lymphoma showed viral localization only in the kidney, the salivary gland, the lung and the stomach by immunohistochemistry and/or endpoint PCR. We conclude that NDV-MLS may be a promising agent for the treatment of lymphomas. Future research is needed to elucidate the optimal therapeutic regimen and establish appropriate biosafety measures.

## 1. Introduction

Treatment of cancer is changing radically with non-chemotherapy-based options rapidly emerging. One growing field is the use of naturally occurring or genetically modified viruses which can both cause direct cell toxicity and oncolysis as well as provoke immune-stimulation with long lasting anti-cancer memory. An extensive number of viruses have been studied as oncolytics both *in vitro* and *in vivo* including small studies in humans [1,2]. One such virus is the Newcastle disease virus (NDV), an avian paramyxovirus that has been shown to have inherent oncolytic and immuno-stimulatory effects. Importantly, NDV can selectively kill cancer cells *in vitro* sparing healthy cells in mammals [2,3,4]. Of note, this virus, especially velogenic (high virulence) strains, is considered a zoonosis, causing mainly conjunctivitis in humans. No infections have been reported in mammals and no human-to-human transmission has been reported [5]. Thus, avian oncolytic NDV could potentially be used to treat cancer safely in different mammals including dogs and humans.

Research on oncolytic viruses has been directed mostly toward solid tumors, which has made limited information available regarding the usefulness of NDV in the treatment of canine or human hematological neoplasias [6,7,8]. Lymphocyte-derived tumors represent the eight and tenth most common cancers in men and women, respectively [9]. Lymphomas are also important causes of disease and death in dogs, making up 7% of all malignant tumors and 83%–90% of the hematological neoplasms [10,11]. Since spontaneous canine and human lymphomas share a similar biological behavior [12,13], dogs represent an excellent opportunity for the performance of translational research studies [10,14,15,16]. Here, we explore the *in vitro* sensitivity of both canine and human lymphoma cells to a lentogenic (low virulence) strain of NDV and report on early tissue biodistribution after intravenous NDV infusion in a dog with spontaneous lymphoma.

## 2. Materials and Methods

### 2.1. Ethical Considerations

Experiments involving animals were carried out under owner-approved written informed consent and were carefully performed by veterinary medical professionals ensuring animal well-being. Guidelines set by the Institutional Animal Care and Use Committee from the Veterinary School at the Universidad Nacional Autónoma de México were followed (Protocol DC-2015-2-5).

### 2.2. Cells and Culture Conditions

The human diffuse large B-cell lymphoma cell line SU-DHL-4 was kindly provided by Mario Vega (Medical Research Unit, Oncology Hospital, Mexican Institute for Social Security). Cells were grown to confluence and sub-cultured every third day before the experiments.

Primary canine lymphoma cells were obtained from a dissected lymph node. The specimen contained >90% neoplastic cells as confirmed by histopathology. Cells were stored frozen at −80 °C in inactivated FBS (Life Technologies, Carlsbad, CA, USA) containing 10% DMSO (Sigma, St. Louis, MO, USA) until use and thawed on the day of the experiment. Human and canine peripheral blood mononuclear cells (PBMC) were isolated from healthy donors (three humans and two dogs). Heparinized blood samples were collected, and PBMC were separated on Ficoll-Paque Plus (GE Healthcare Bioscience, Marlborough, MA, USA) by density gradient centrifugation. Mononuclear cells were subsequently isolated from the interphase and cultured. Human blood samples were collected 24 h before experiments and processed as mentioned above, while canine mononuclear cells were collected and processed on the day of the experiments. Cells were cultured in RPMI-1640 medium (Sigma, St. Louis, MO, USA) containing 10% inactivated FBS (Life Technologies) and antibiotics (100 IU penicillin/mL and streptomycin 100 mg/mL, (Life technologies)) at 37 °C in a humidified atmosphere of 5% CO_2_.

Vero cells (ATCC CCL81) were cultured with DMEM medium (Life Technologies) plus 10% inactivated FBS (Life Technologies) and antibiotics (100 IU penicillin/mL and streptomycin 100 mg/mL, (Life technologies)). Cells were grown to confluence and sub-cultured every third day before the experiments. These cells were used to determine tissue culture infective dose (TCID_50_) by the Reed-Munch method [17].

### 2.3. Newcastle Disease Virus

**Figure 1:**
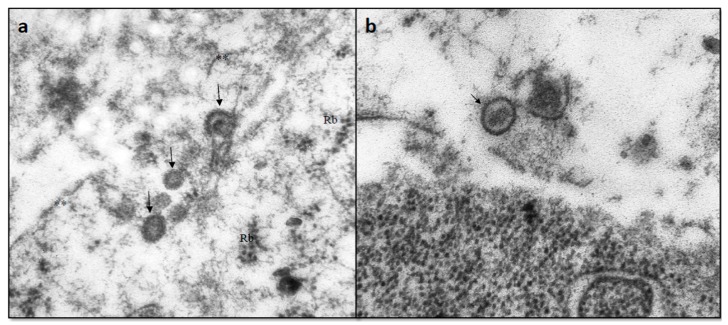
Transmission electron microscopy of diffuse large B-cell lymphoma cells studied *ex vivo* after intratumoral injection of NDV-MLS (**a**,**b**). Viral particles (150 nm) are immersed in the cytoplasm and show characteristic electronlucent short projections on the surface (Black arrows). Uranyl acetate and lead citrate contrast technique. 50,000× magnification.

NDV-MLS is a naturally attenuated (non-recombinant) virus derived from LaSota strain, grown in pathogen-free embryonated chicken eggs (ALPES, Puebla, Mexico). NDV-MLS produces a mean death time (MDT) in chick embryos of greater than 90 h, suggesting that it remains a lentiviral strain [18]. When NDV-MLS is pre-activated with trypsine, it induces a characteristic small syncytia formation on African green monkey kidney cells (Vero cells) [19]. No cytotoxic effect is seen when Madin-Darby bovine kidney cells (MDBK cells) are exposed to the virus, as expected for most lentiviral strains [20]. We have also documented that NDV-MLS is able to infect malignant lymphoma *in vivo* in dogs as confirmed by electron microscopy (Figure 1).

Viral replication was carried out by inoculation of specific pathogen-free chicken eggs with 0.1 mL of NDV-MLS (10^8^ TCID_50_/mL). After 3 days, allantoic fluid was removed from the eggs aseptically, and the presence of the virus was confirmed by micro-haemagglutination using chicken red blood cells. Batches of NDV-MLS used in this study were tested for the presence of microbiological agents through cultures. Allantoic fluid protein concentration by Kjeldahl analysis yielded counts below 0.05%. Aliquots were stored at −80 °C until use. NDV-MLS infectivity was quantified using a tissue culture infective dose (TCID_50_) in Vero cells by the Reed-Munch method [17]. Briefly, Vero cells were cultured in 96-well plates (Nunclon™, Kamstrupvej, Denmark) until confluence was attained. Serial 10-fold dilutions, previously incubated for 2 h at 37 °C with 0.3 μL/mL of trypsin, were added to Vero cell monolayers and incubated at 37 °C in 5% CO_2_ until there was no further cytopathic effect after consecutive daily monitoring. Cell monolayers were then fixed with 100% methanol and stained with 0.2% crystal violet. Wells with cytopathic effect were recorded.

### 2.4. Cytotoxicity Assay

Cells were plated (2 × 10^5^/well) in duplicate or triplicate in 48-well plates (Nunclon™) and incubated with either NDV-MLS at a multiplicity of infection (MOI) of 100 or with virus-free allantoic fluid. A MOI of 100 was chosen based on previous dose-response experiments that showed a consistent cytotoxic effect. Cells were harvested after 72 h, and viability was measured using a vital trypan-blue exclusion assay [21] as well as by flow cytometry. Cell viability was normalized uninfected well and expressed as the mean percentage of viable cells ± SEM (standard error of the mean). As positive control for cytotoxicity, we used a combination of chemotherapy drugs, which make up the standard treatment regimen for both human and canine diffuse large B cell lymphomas. The regimen is known as CHOP. Concentrations were established maintaining the ratio between drugs. Cells were exposed to cyclophosphamide (10.08 μg/2 × 10^5^ cells per mL), hydroxydaunorubicin (0.0672 μg/2 × 10^5^ cells per mL), oncovin (0.0188 μg/2 × 10^5^ cells per mL), and prednisone (0.05376 μg/2 × 10^5^ cells per mL). After evaluating different doses, the concentration of CHOP that produced 80% cell death at 72 h was chosen for further experiments.

### 2.5. Flow Cytometry

Viability of lymphoma cells and peripheral blood mononuclear cells from both humans and dogs was assessed by two-color flow cytometry on a FACSCanto flow cytometer (Becton Dickinson, Franklin Lakes, NJ, USA). Cells were harvested 72 h post-exposure to allantoic fluid alone or NDV-MLS at a MOI of 100, washed twice with PBS buffer, quantified, and then incubated 15 min at room temperature in the dark with propidium iodide (PI) and allophycocyanin (APC)—conjugated Annexin V antibody in Annexin binding buffer according to the manufacturer´s instructions (Annexin V Apoptosis Detection Kit APC, Affymetrix eBioscience, San Diego, CA, USA). Additionally, healthy human B-cells were identified by immunophenotype with a CD19-FITC stain. Analysis of flow cytometry data was performed using the FlowJo 9.0 software (FLOWJO, LLC: Ashland, OR, USA). Final yield values were calculated on the basis of specific population frequencies within each condition.

### 2.6. Spontaneous Canine Lymphoma and Early NDV-MLS in Vivo Distribution

NDV has been reported as well tolerated in human cancer patients and healthy non-human primates [22,23,24,25]. We studied early biodistribution of NDV-MLS *in vivo* after intravenous infusion of a single dose of 1 × 10^12^ TCID_50_ in a canine patient with end-stage incurable spontaneous aggressive T-cell lymphoma with the owner's informed consent. The dose of NDV-MLS was calculated based on previous human clinical trial experience with oncolytic viruses [23,24,25]. Lymph node and bone marrow biopsies were performed prior to viral infusion and fixed in 10% formalin, paraffin-embedded, and sectioned for review by the hemato-pathologists. Sections were stained with hematoxylin-eosin (H & E) and with monoclonal mouse anti-human CD79a (Spring Bioscience Corp., Pleasanton, CA, USA) and anti-CD3 antibodies (Dako, Carpinteria, CA, USA) as B-cell and T-cell markers, respectively.

The dog was hydrated and medications were administered to prevent pain and nausea. The dog received meloxicam (an enolic acid that blocks cyclooxygenase), tramadol (a narcotic-like analgesic mu-opioid receptor agonist that blocks the reuptake of norepinephrine and serotonin), ondansetron (a serotonin 5-HT3 antagonist), and ranitidine as a gastric mucosa protector. There are no reports of these drugs interfering with viral distribution, yet this possibility cannot be completely ruled out. Although no mammal-human transmission has been documented, standard security measures for owners and the medical team were followed.

### 2.7. Post-Mortem Viral Biodistribution Tissue Analysis

Necropsy was carried out after the natural death of the dog, which occurred 24 h post NDV-MLS infusion. Organ samples were fixed in 10% formalin or stored frozen (−80 °C) without additives. Paraffin-embedded samples of distinct organs were sectioned and stained with hematoxylin and eosin (H & E). Immunohistochemistry for NDV (anti-hemagglutinin and neuraminidase (HN) protein, antibodies-online Inc., Atlanta, GA, USA) was performed using immunoperoxidase according to the manufacturer’s recommendations. Tissue sections were cut) and slides were boiled in a 0.01 M sodium citrated buffer, pH 6.0 at 99–100 °C for 15–20 min. Slides were then removed from heat and allowed to stand in buffer for 20 min. Subsequently, sections were rinsed twice with Tris-Buffered Saline with Tween (TBST) for 5 min at room temperature (RT). Endogenous peroxidase was blocked with 3% H_2_O_2_ for thirty minutes. A universal protein block was applied (5% normal goat serum) for two hours at room temperature (RT) and subsequently incubated with diluted polyclonal rabbit anti-NDV antibody (antibodies-online Inc., Atlanta, GA, USA) overnight at 4 °C (5% normal goat Rabbit IgG was used as isotype control). Slides were rinsed twice with TBST, and the secondary antibody was added for 2 h at RT with gentle agitation, rinsed twice with TBST, developed with chromogen for 10 min at RT and washed in distilled water for 1 min. NDV-MLS infected chicken embryo tissue was used as positive control, and non-infected chicken embryo tissue as well as canine health lymph node biopsies were used as negative controls. Blood and cerebrospinal fluid were also collected for further RNA isolation. Serum, cell pellets and cerebrospinal fluid were stored at −80 °C without additives.

### 2.8. RNA Isolation and Reverse Transcription PCR (RT-PCR)

Tissues, cell pellets, serum and cerebrospinal fluid were processed according to the manufacturer’s instructions to isolate RNA from solid or liquid samples, respectively (RNeasy Mini Kit/QIAamp Viral RNA Mini Kit, QIAGEN, Valencia, CA, USA). The primers used for end-point RT-PCR were generated by the alignment of published conserved NDV nucleotide sequences, representing a fragment of 527 bp of the nucleoprotein (NP) gene (Table 1). For reverse transcription (RT), 5 μL of extracted nucleic acid was added to the following reaction mix: 2 μL of 10 mM oligos, 0.5 μL of 10 mM dNTP mix, 2 μL of buffer, 0.5 μL of polymerase, and 0.2 μL of RNase inhibitor for a total reaction volume of 10.2 μL. As positive control, 5 μL of NDV-MLS-extracted nucleic acid was used. For negative control, only sterile water was added. The reaction was subsequently incubated in a thermocycler under the following conditions: 50 °C for 30 min and 95°C for 15 min for RT; for PCR, 94 °C for 2 min, followed by 35 cycles at 94 °C for 30 s (denaturation), 55 °C for 30 s (annealing), and 72 °C for 1 min (extension). Samples were subsequently chilled at 4 °C and run on 2% agarose E-gel^®^ (Thermo Fisher Scientific, Waltham, MA, USA) and photographed.

**Table 1:** Primers used for detection of NDV by end-point RT-PCR.

Gene	Primer Sequence	Lengh of Product (bp)
NP	FP: 5′-ACCAAACAGAGAATCCGTGAGTTACGATAA-3′	527
	RP: 5′-GGAGAGATCCTGCTATCATCGCAAATCT-3′	

NP: Nucleoprotein; FP: Forward primer; RP: Reverse primer.

Total RNA was extracted from liquid samples and amplified in multiplex reactions using the StepOnePlus™ Real-Time PCR System (Applied Biosystems, Foster City, CA, USA) and NDV internal positive control-specific primer/probe combinations (FIND-IT Newcastle Kit, BioTecMol, Mexico City, Mexico). Each sample was treated with lysis buffer plus the carrier included in the RNeasy Mini Kit/QIAamp Viral RNA Mini Kit (QIAGEN). RNA was extracted according to the manufacturer’s instructions. NDV-specific quantitative real-time reverse transcription PCR was performed using 10 μL RNA in a StepOnePlus™ Real-Time PCR System (Applied Biosystems) using TaqMan NDV Virus 1-Step RT-PCR-TR 2× (BioTecMol) in a final volume of 20 μL. Cycling conditions were as follows: 30 min at 42 °C, 10 min at 95 °C (40 cycles of 15 s at 95 °C) and 45 s at 60 °C.

### 2.9. Statistical Analysis

The Prism (version 6.0, GraphPad, La Jolla, CA, USA) software was used for statistical analysis. Data were expressed as mean ± SEM of independent experiments. Ordinary one-way ANOVA with Bonferroni’s or Tukey’s multiple comparison tests was used to compare groups.

## 3. Results

### 3.1. Malignant Lymphocytes of Human and Canine Origin Are Selectively Killed by the NDV-MLS

**Figure 2:**
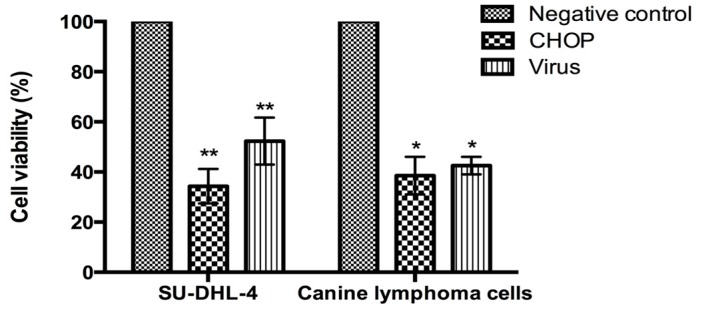
Cytotoxicity of NDV-MLS on human malignant lymphoma (SU-DHL-4) and primary canine lymphoma-derived cells. Viability was assayed by trypan blue at 72 h post-infection. The figure shows the mean percentage cell viability (± SEM) of five independent experiments. Significant reduction in cell viability was seen with viral exposure for both human and canine lymphoma cells. * *p* < 0.05; ** *p* < 0.001.

We first investigated whether the NDV was able to induce death of malignant lymphocytes of human and canine origin *in vitro*. We carried out cytotoxicity assays on the SU-DHL-4 human lymphoma cell line and on primary canine lymphoma cells using either NDV-MLS at a MOI (multiplicity of infection) of 100 or CHOP chemotherapy (cyclophosphamide, hydroxydaunorubicin, oncovin and prednisone). Cell viability was analyzed by trypan blue exclusion assay 72 h post-infection and by flow cytometry. A cytotoxic effect was observed in both cell types (Figure 2). In the canine lymphoma cells, virus treatment decreased viability to the 43% ± 3.5% while CHOP treatment decreased it to the 39% ± 7.5%. SU-DHL-4 cells treated with the virus showed a decrease in viability to the 52% ± 9.4%, while CHOP treatment decreased viability to 34% ± 6.9%.

The oncolytic effect of NVD-MLS in both cell types was confirmed by multi-parametric flow cytometry using double staining with Annexin V-APC (allophycocyanin) and propidium iodide (PI). Only Annexin V and PI double-negative cells were considered viable. Again, considerable decrease in cell viability was observed in both cell types infected with the virus: 41.2% ± 5.9% and 59.5% ± 14.8% in canine and human cells, respectively * *p* < 0.05. Cell viability with control CHOP decreased to 12.7% ± 1.1% and 10.1% ± 3.1%, respectively.

**Figure 3:**
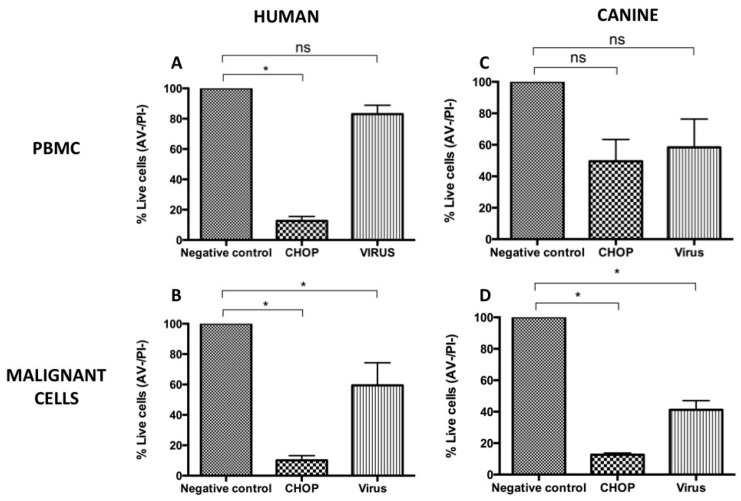
Effect of NDV-MLS treatment on the cell viability of human (**A**) PBMC and (**B**) SU-DHL-4 cells as well as canine (**C**) PBMC and (**D**) lymphoma cells at 72 h post-infection. Cells were subjected to Annexin V/PI staining and analyzed by flow cytometry. Quantitative analysis of Annexin V/PI negative cells is shown. Results are representative of five independent experiments and show a significant cytotoxic effect on both human *p* < 0.001 and canine lymphoma cells * *p* < 005, ns: non-significant.

### 3.2. Peripheral Blood Mononuclear Cells Are Less Sensitive to the NDV-MLS

Oncolytic viruses are known to have preferential cytolytic effects over cancer cells [26]. Likewise, NDV has been shown to possess a selective capacity for killing cancer cells derived from solid tumors over healthy cells. We investigated whether NDV was preferentially cytotoxic for lymphoma cells. Infected, healthy human and canine PBMC were subjected to double staining multi-parametric flow cytometry analysis using Annexin V-APC/PI. Human malignant lymphocytes (SU-DHL-4) were more sensitive to the NDV cytotoxicity than their healthy counterparts with a decrease on cell viability to 59.5% ± 25.7%, and 83.1% ± 10.1%, respectively. In dogs there was a trend for preferential cytotoxicity of canine malignant *versus* PBMC 41.3% ± 8.3% and 58.4% ± 25% (Figure 3).

**Figure 4:**
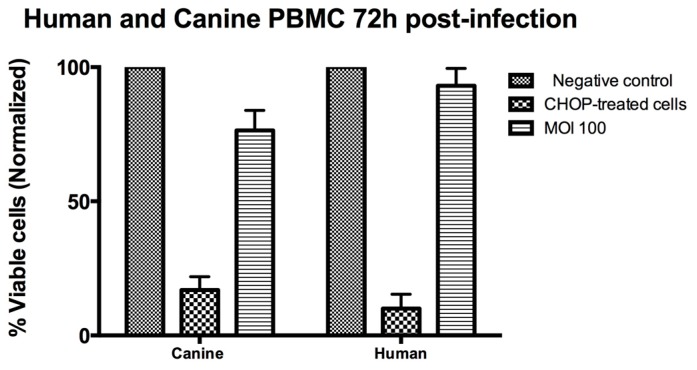
Effect of NDV-MLS (MOI 100) treatment on cell viability of healthy human and canine PBMC. Flow cytometry was carried out 72 h post-infection. Only cells negative for both Annexin V and propidium iodide staining were considered viable. Bars represent results from two independent experiments.

The sensitivity of canine PBMC to NDV-MLS is variable. In experiments comparing canine and human PBMC, we again found NDV-MLS to be non-toxic for healthy human cells, while canine PBMC are consistently affected by viral exposure, with a mean decrease in survival of 20% (Figure 4). Inter-individual variability may be responsible for this finding since blood from different dogs was used.

### 3.3. Human Non-Malignant B-Cells Are Not Sensitive to the Cytotoxic Effect of the NDV-MLS

**Figure 5:**
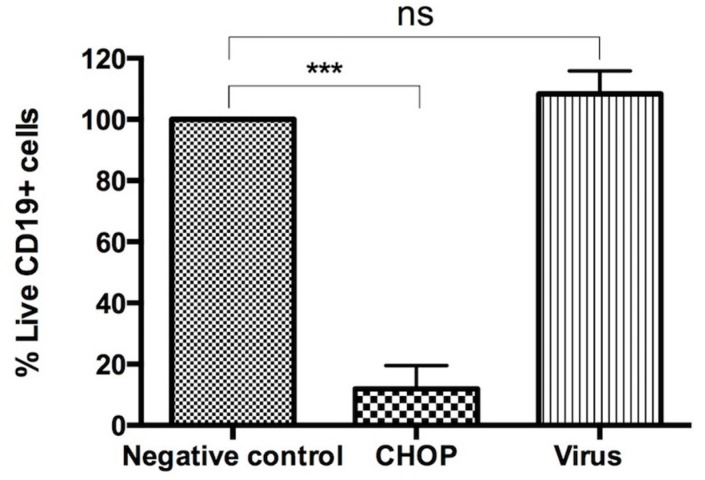
Effect of NDV-MLS treatment on healthy human CD19 positivecells. At 72 h post-infection, cells were subjected to Annexin V/PI/CD19-FITC staining and analyzed by flow cytometry. Quantitative analysis of apoptotic CD19 cells is shown. Bar graph depicts viable (Annexin V and PI negative) CD19 positive B-cells. Results are representative of three independent experiments. Healthy B-cells did not show decreased cell survival upon viral exposure, but were highly sensitive to CHOP chemotherapy. *** *p* < 0.0001 ns: non-significant.

Since NDV-MLS was found to be toxic to malignant B lymphocytes, we explored the ability of NDV-MLS to lyse healthy human B-lymphocytes from PBMC. Cells were subjected to a triple staining multi-parametric flow cytometry analysis using Annexin V-APC/PI/CD19-FITC. As expected, viability decreased markedly in normal B lymphocytes treated with CHOP, while there was no effect on healthy B-lymphocytes upon viral exposure. B-lymphocyte viability was 12% ± 7.6% when treated with CHOP, and 108.3% ± 7.5 when exposed to NDV-MLS (Figure 5).

### 3.4. Early Biodistribution Is Not Widespread

**Figure 6:**
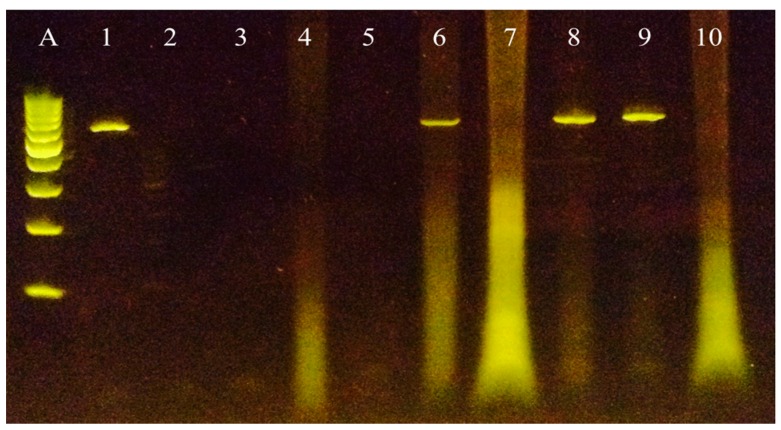
Endpoint RT-PCR from canine tissues (post-mortem). The amplification of a band of 527 base pairs, corresponding to a fragment of the *NP* gene of NDV is shown. Lane (**A**) 100 bp ladder, lane 1: positive control (RNA from NDV-MLS strain), lanes 2 and 3: negative controls (sterile water), lanes 6, 8 and 9: positive gastric, renal and salivary gland tissues, respectively.

We analyzed the early biodistribution 24 h post-intravenous injection of 1 × 10^12^ TCID_50_ NDV-MLS in a spontaneous canine lymphoma patient. The dog had end-stage T-cell lymphoma confirmed by CD3 immunohistochemical-positive staining. Post-mortem tissue samples from all organs were evaluated for the presence of viral RNA by reverse transcription polymerase chain reaction (RT-PCR). Only salivary gland, gastric, and renal tissues were positive (Figure 6). Low-level infection of tissues in which viral RNA was not amplified cannot be excluded.

**Figure 7:**
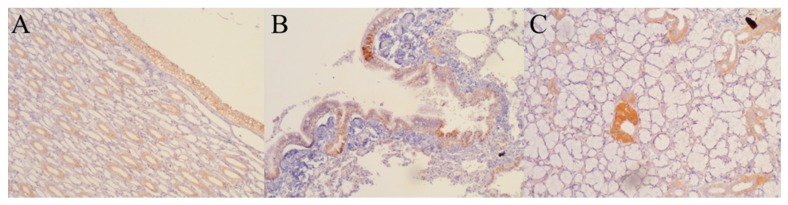
Immunohistochemistry (IHC) with a polyclonal rabbit antibody against viral hemagglutinin-neuraminidase (H-N) protein was performed to detect NDV in tissues. Positive staining (brown) was seen in (**A**) kidney; (**B**) lung, and (**C**) salivary gland (10× magnification).

No gross pathological changes were observed in the non-cancerous tissues. In order to confirm the presence of viral proteins in tissues, immunohistochemistry was performed. We found positive staining for viral nucleoprotein (NP) in only three healthy tissues (lung, salivary gland and kidney), but malignant lymphoma did not express NP viral protein. (Figure 7, Table 2).

**Table 2:** Post-mortem tissue analysis. All tissues enlisted were tested by RT-PCR and IHC for the presence of virus. Positive (+) and negative (-) tissue samples are shown.

Tissue	RT-PCR	IHC	Tissue	RT-PCR	IHC
Kidney	+	+	Pancreas	−	−
Salivary gland	+	+	Adipose tissue	−	−
Stomach	+	−	Colon	−	−
Lung	−	+	Brain	−	−
Cancerous tissues	−	−	Cerebelum	−	−
Striated skeletal muscle	−	−	Adrenal gland	−	−
Fibroadipose tissue	−	−	Cell package	−	−
Nervous package	−	−	Serum	−	−
Liver	−	−	Cerebrospinal fluid	−	−
Squamous mucosa	−	−			

Regarding the secretion of NDV-MLS in body fluids, preliminary experiments do show viral RNA in urine by qRT-PCR). This may be a relevant safety issue despite the use of a lentiviral strain. We are currently obtaining further data related to viral replication and biosafety.

## 4. Discussion

The use of oncolytic viruses has been previously reported with the measles virus showing promising potential for the treatment of acute and chronic lymphoid leukemias *in vitro* and of myeloma *in vivo,* as well as with adenovirus D *in vitro* lymphoma and chronic lymphocytic leukemia experiments and in a murine model of lymphoma [27,28,29]. The Newcastle disease virus has been proposed as a promising therapeutic agent because of its ability to preferentially kill cancer cells [5]. Previous published data have shown the oncolytic potential of LaSota strain wild type or genetically modified in solid tumors *in vitro* [30,31,32]. A genetically modified LaSota strain has been tested in leukemic blasts and myeloma cell lines [4,33], but, to our knowledge, there is no information about the use of lentiviral strains in aggressive lymphomas

In this paper, we report *in vitro* work with a low virulence (lentogenic) strain NDV-MLS that is naturally attenuated and is non-recombinant. NDV-MLS was studied *in vitro* in both canine primary lymphoma-derived cells and in a human cell line of aggressive diffuse large B-cell lymphoma, as well as in healthy peripheral blood mononuclear cells.

We found that NDV-MLS is preferentially cytotoxic for malignant B-cells of human and canine origin. B-cell-derived lymphomas are the most prevalent subtypes in both species [34,35,36,37]. Two previous studies have also reported susceptibility of human lymphoma B-cells *in vitro* (Burkitt Daudi cells) to other non-modified lentogenic and mesogenic NDV strains [6,7]. On the other hand, we demonstrated a differential cytotoxic effect upon healthy mononuclear cells. Selectivity of NDV replication in human cancer cells is given largely by the poor response of these cells to type I interferon (mainly β) production, making them incapable of inducing a rapid and sustained production of antiviral proteins [38,39]. Cytotoxic selectivity has also been attributed to the overexpression of proteins such as Bcl-xL, for example, in A540 cells (lung cancer), in which the oncolytic effect of the virus increases [40]. We found selectivity of NDV-MLS for lymphoma cells of both human and canine origin over healthy mononuclear cells and normal B-cells, a finding that is relevant for future *in vivo* use.

The benefit of using an avian virus to treat cancer in mammals is the lack of previous exposure to the virus and thus of neutralizing antibodies. The convenience of a lentogenic strain is that handling of the virus is safer and avoids the risk of causing an outbreak in avian hosts. The main advantage is being able to treat human patients and other mammals with potential benefits for both human and veterinary medicine [11,41,42,43,44,45]. Lymphomas are frequent and still a therapeutic challenge in both human and canine species.

Oncolytic viruses have been studied as a therapeutic alternative to canine cancer, with favorable preclinical results [8,46,47]. To date, only vesicular stomatitis virus is being tested in dogs with a myeloma, a hematological malignancy [48]. The NDV had never been tested in canine cancer. In a recent study of I.V. administration of recombinant NDV LaSota strain in healthy non-human primates, escalating doses from 1 × 10^7^ to 1 × 10^9^ embryo infectious dose (EID_50_) or TCID_50_ were safely administered [22]. Regarding reports in humans, non-recombinant NDV has been given to patients with advanced and/or metastatic solid cancers [23,24]. In a set of patients treated with NDV-PV107, derived from the MK107 vaccine strain, the authors reported the inclusion of three patients with lymphoma, yet they did not report the results observed in these patients in particular [23]. Acute reactions (principally chest and back pain non-cardiogenic, and hypertension) occurred within 5 min of the onset of treatment and resolved spontaneously within half an hour [23,24]. We found that intravenous administration of 10^12^ TCID_50_ of NDV-MLS had limited early virus dissemination to normal tissues. There is no published data regarding bio-distribution in spontaneous lymphoma. We found positive immunohistochemical staining for the NDV only in salivary glands, renal and gastric tissue, reflecting a possible low toxicity potential to normal tissues in sick individuals. We did not find viral particles in tumor tissue at 24 h after intravenous infusion; considering that NDV-MLS is classified as a lentogenic strain, it is possible that longer times will be required for viral dissemination into tumor tissue.

## 5. Conclusions

We conclude that NDV-MLS, a non-pathogenic strain for mammals, is a promising agent for the treatment of human and canine diffuse large B cell lymphomas. Future research is needed to elucidate the optimal therapeutic regimen and to establish the appropriate biosafety measures in order to prevent environmental spread.
